# Supplementary material for: Smart Drug-Delivery System of Upconversion Nanoparticles Coated with Mesoporous Silica for Controlled Release
Source: Pharmaceutics. 2022 Dec 27;15(1):89. doi: 10.3390/pharmaceutics15010089 (PMC9864186; doi:10.3390/pharmaceutics15010089)
Supplement: Supplementary file 1 [file pharmaceutics-15-00089-s001.zip › pharmaceutics-2081617-supplementary.pdf]

pharmaceutics

Electronic Supplementary Material (ESI) for Pharmaceutics

Smart Drug-Delivery System of Upconversion Nanoparticles Coated with Mesoporous Silica for Controlled Release

Yanan Huang [1,2], Ziqing Du [1], Guochen Bao [1], Guocheng Fang [1], Matthew Cappadona [1], Lana McClements [3], Bernard E. Tuch [4,5], Hongxu Lu [6,*] and Xiaoxue Xu [1,7,*]

1. School of Mathematical and Physical Sciences, Faculty of Science, University of Technology Sydney, Sydney, NSW 2007, Australia
2. Department of Chemistry, Shanghai Key Lab of Molecular Catalysis and Innovative Materials, iChEM, Fudan University, Shanghai 200437, China
3. School of Life Sciences & Institute for Biomedical Materials and Devices, Faculty of Science, University of Technology Sydney, Sydney, NSW 2007, Australia
4. Australia Foundation for Diabetes Research, Sydney, NSW 2007, Australia
5. Department of Diabetes, Central Clinical School, Faculty of Medicine, Nursing & Health Sciences, Monash University, Melbourne, VIC 3800, Australia
6. State Key Laboratory of High Performance Ceramics and Superfine Microstructure, Shanghai Institute of Ceramics, Chinese Academy of Sciences, 1295 Dingxi Road, Shanghai 200050, China
7. School of Biomedical Engineering, Faculty of Engineering and Information Technology, University of Technology Sydney, Sydney, NSW 2007, Australia

* Correspondence: hongxu.lyu@mail.sic.ac.cn (H.L.); xiaoxuehelen.xu@uts.edu.au (X.X.)

Citation: Huang, Y.; Du, Z.; Bao, G.; Fang, G.; Cappadona, M.; McClements, L.; Tuch, B.E.; Lu, H.; Xu, X. Smart Drug-Delivery System of Upconversion Nanoparticles Coated with Mesoporous Silica for Controlled Release. *Pharmaceutics* **2023**, *15*, 89. https://doi.org/10.3390/pharmaceutics15010089

Academic Editor: Avi Domb

Received: 22 November 2022
Revised: 19 December 2022
Accepted: 23 December 2022
Published: 27 December 2022

Copyright: © 2022 by the authors. Submitted for possible open access publication under the terms and conditions of the Creative Commons Attribution (CC BY) license (https://creativecommons.org/licenses/by/4.0/).

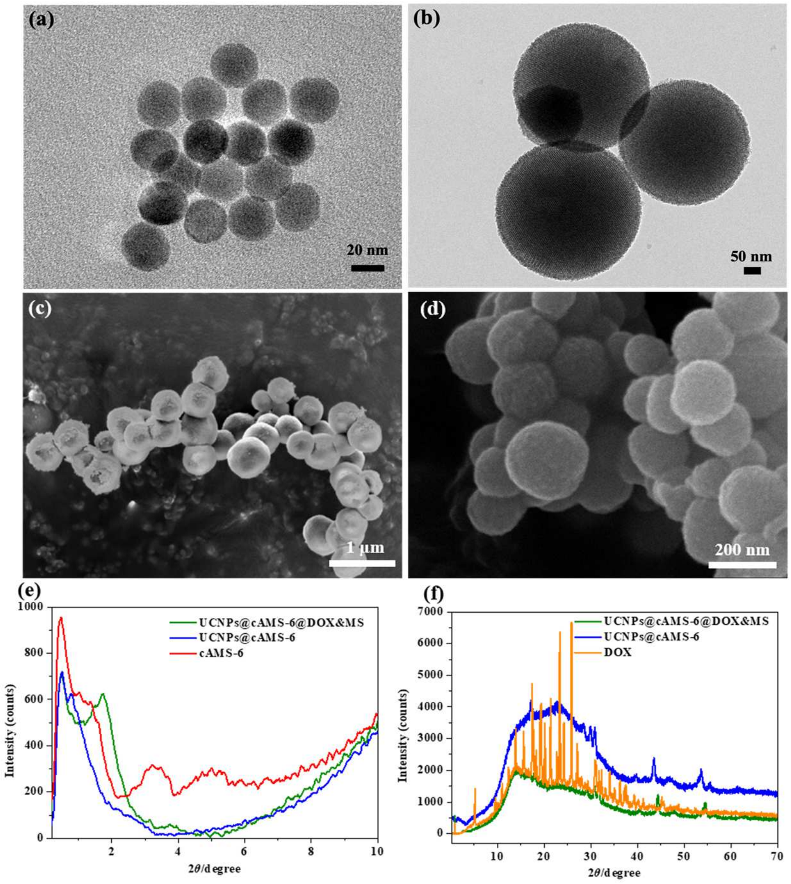

Figure S1. Properties of mesoporous particles of AMS-6, UCNPs@cAMS-6 and UCNPs@cAMS-6@DOX&MS. (**a**) Representative TEM image of the synthesized UCNPs. (**b**) representative TEM

images of cAMS-6. (**c**) SEM images of the cAMS-6 and (**d**) UCNPs@cAMS-6 nanoparticles, respectively. (**e**) Small-angel X-ray diffraction patterns of cAMS-6, UCNPs@cAMS-6 and UCNPs@cAMS-6@DOX&MS showing mesoscale diffraction peaks associated with the cubic mesostructure (a_0 = 116.5 Å for the calcined AMS-6). (**f**) Powder X-ray diffraction patterns of UCNPs @cAMS-6@DOX&MS compared to the pure Dox compound showing the absence of scattering peaks from crystalline drug.

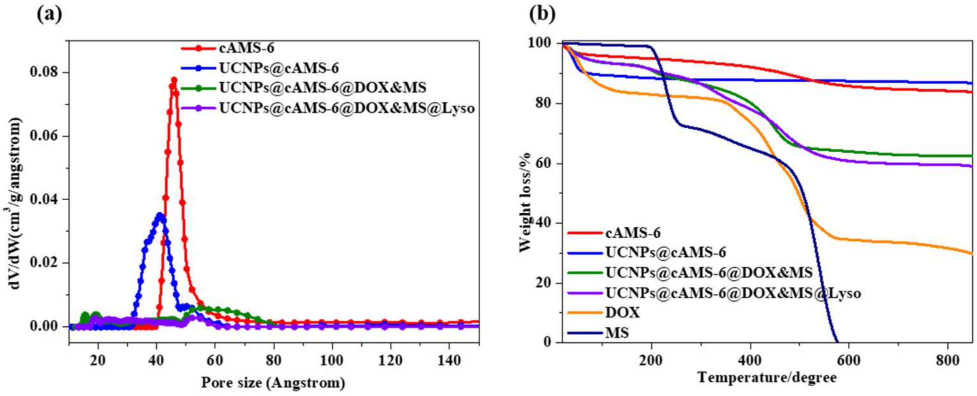

Figure S2. Drug loading and Protein Lysozyme coated UCNPs@cAMS-6. (**a**) Pore size distribution curve of cAMS-6, UCNPs@cAMS-6, UCNPs@cAMS-6@Dox/MS and UCNPs@cAMS-6@Dox/MS @Lyso samples. (**b**) Thermogravimetric analysis curves of cAMS-6, UCNPs@cAMS-6, UCNPs@cAMS-6@Dox/MS, UCNPs@cAMS-6@Dox/MS@Lyso, Dox and Molecule stirrer.

Table S1. Textural parameters of cAMS-6, UCNPs@cAMS-6, UCNPs@cAMS-6@Dox&MS and UCNPs@cAMS-6@Dox&MS@Lyso. The average pore volume, average pore size and surface area (P_{vol}, P_{size} and S_{Area}, respectively) are obtained from N_2 adsorption measurements. The drug and Lysozyme content (%wt loss) was determined by thermogravimetric analysis. Data for Lysozyme coated particles was measured after washing unbound proteins (see supporting information experimental details).

**Sample**	**P_{vol} (cm^3·g^{-1})**	**P_{size} (Å)**	**S_{area} (m^2·g^{-1})**	**%wt Lost**
cAMS-6	0.77	46.50	694.96	8.3
UCNPs@cAMS-6	0.35	46.33	446.54	13.0
UCNPs@cAMS-6@Dox&MS	0.19	30.93	240.39	37.5
UCNPs@cAMS-6@Dox&MS@Lyso	0.11	25.57	156.12	41.31

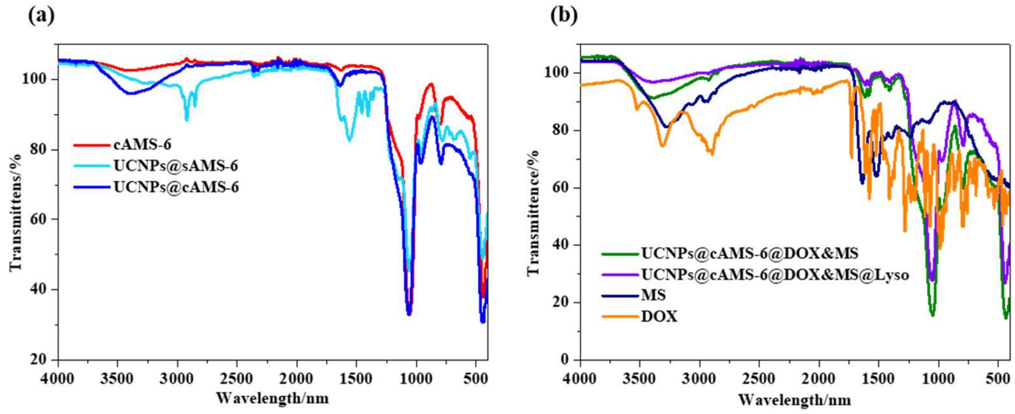

Figure S3. Fourier Transform Infrared (FT-IR) Spectra of (**a**) cAMS-6, UCNPs@sAMS-6, UCNPs@cAMS-6, and (**b**) UCNPs@cAMS-6@Dox&MS and UCNPs@cAMS-6@Dox&MS@Lyso samples. For comparison pure Dox and MS are also shown.

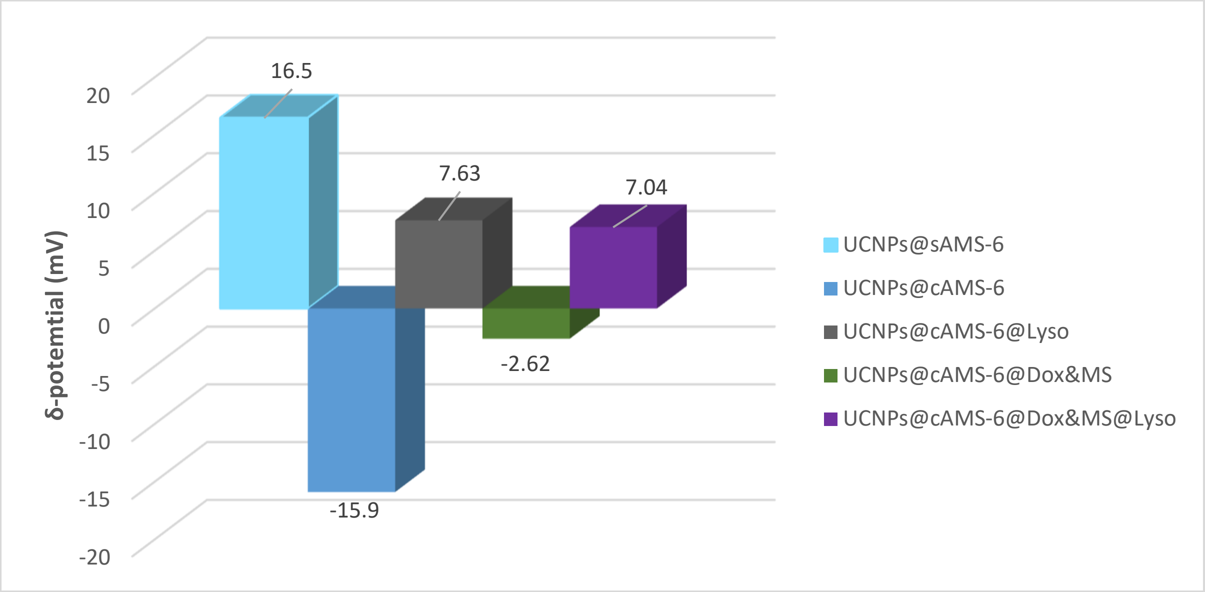

Figure S4. The zeta potential of UCNPs@sAMS-6, UCNPs@cAMS-6, UCNPs@cAMS-6@Lyso, UCNPs@cAMS-6@Dox&MS and UCNPs@cAMS-6@Dox&MS@Lyso samples.

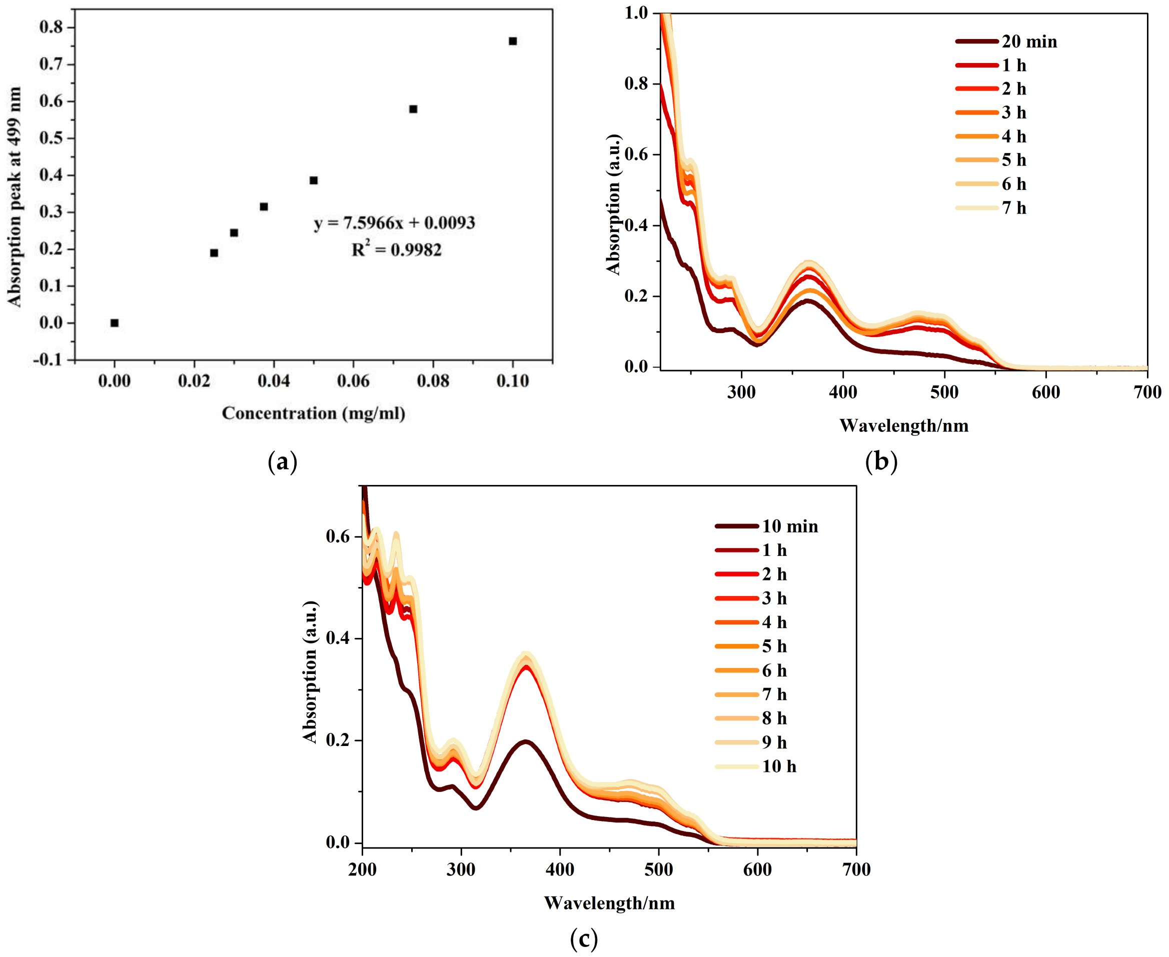

Figure S5. (**a**) Standard calibration curve of Dox at 499 nm; UV-Vis absorption profile of Dox absorption test in (**b**) UCNPs@cAMS-6@Dox&MS and (**c**) UCNPs@cAMS-6@Dox&MS@Lyso samples.

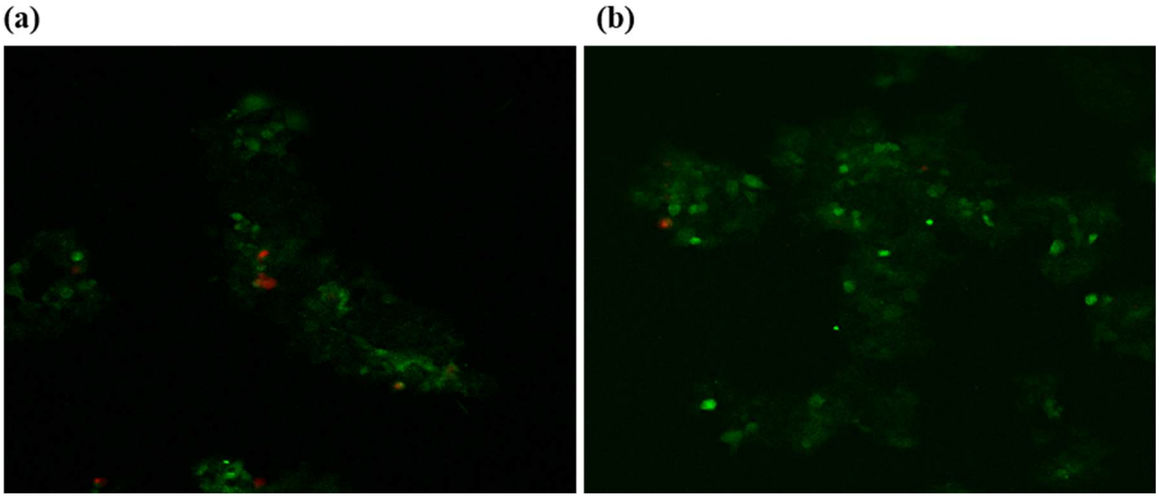

Figure S6. Confocal microscopic images of live (green) and dead (red) cells exposed to (**a**) UCNPs@cAMS-6 and (**b**) UCNPs@cAMS-6@Lyso. All images were taken under 10× objective lens.

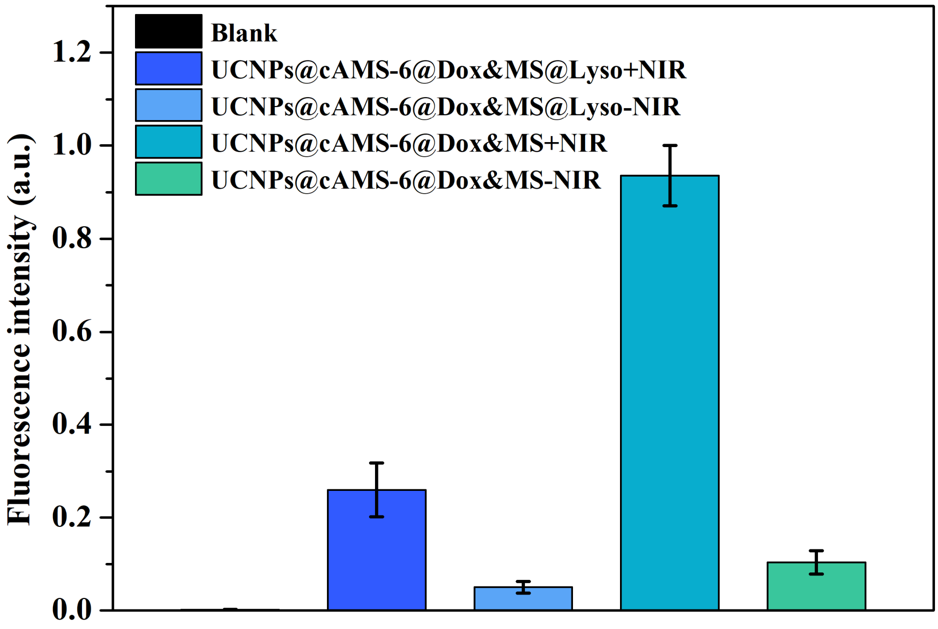

Figure S7. Fluorescence intensity in response to controlled release of Dox in all prepared samples.
